# Impact of the COVID-19 Pandemic on Family Caregivers

**DOI:** 10.1093/geroni/igaa057.3475

**Published:** 2020-12-16

**Authors:** Tina Kilaberia, Janice Bell, Kristen Bettega, Jennifer Mongoven, Kathleen Kelly, Heather Young

**Affiliations:** 1 UC Davis Health, Sacramento, California, United States; 2 University of California Davis, Sacramento, California, United States; 3 Betty Irene Moore School of Nursing at UC Davis, Sacramento, California, United States; 4 University of California Davis Health, Sacramento, United States; 5 Family Caregiver Alliance, San Francisco, California, United States

## Abstract

The COVID-19 pandemic has affected human life in unprecedented ways. Lives of older persons and their families have been especially adversely affected. Eleven Caregiver Resource Centers (CRCs) support family caregivers across the state of California, providing services such as assessment, counseling and respite. This presentation is part of a larger evaluation study and addresses the impact of the COVID-19 pandemic on family caregivers and systems supporting them. We interviewed directors, clinical staff and family consultants (n=35) from CRCs across diverse communities and geography in California, conducting semi-structured focus group interviews by Zoom. Questions explored perceptions of staff about effects on caregivers and implications for systems of support for caregivers. Interviews were transcribed and analyzed using the Dedoose software. Caregiver effects included increased isolation, higher stress, loss of support, neglected health needs, and accelerated technology adoption. Caregivers at particular risk were those facing multiple demands and experiencing compromised resources under the pandemic, such as closure of adult day care. System effects included challenges with hastened virtual delivery, disruption in services, and new opportunities to serve clients virtually. Community resources, such as internet connectivity, exacerbated disparities for family caregivers. We make recommendations to mitigate these challenges including technology platforms to support service delivery and education, training and preparedness for both caregivers and providers. These recommendations are relevant to the ongoing COVID-19 pandemic and adaptable to other crisis situations such as natural disasters.

